# Evaluation of clinical trials by Ethics Committees in Germany – results and a comparison of two surveys performed among members of the German Association of Research-Based Pharmaceutical Companies (vfa)

**DOI:** 10.3205/000206

**Published:** 2015-01-27

**Authors:** Hagen Russ, Susanne Busta, Bertfried Jost, Thomas D. Bethke

**Affiliations:** 1 Lilly Deutschland GmbH, Bad Homburg, Germany; 2 Bristol-Myers-Squibb GmbH & Co. KGaA, Munich, Germany; 3 Boehringer Ingelheim GmbH, Ingelheim, Germany

**Keywords:** Ethics Committees, application procedure, formal and content-related objections, clinical trials

## Abstract

**Objective: **The objective of this project was to evaluate the quality and quantity of initial applications for a clinical trial according to § 7 of the German Good Clinical Practice (GCP) ordinance (German: GCP-Verordnung, GCP-V), the quality of evaluations of those applications by Ethics Committees (ECs)/Investigational Review Boards (IRBs) in Germany as well as the pattern of EC objections in their reasoned opinions (vote). In order to identify a change over time, the results of the present survey were also compared with a survey performed in 2008.

**Methods: **Based on reasoned opinions issued by the respective EC in charge of the coordinating principle investigator (coordinating EC) in 2011, a written survey among members of the German Association of Research-Based Pharmaceutical Companies (vfa) was conducted in 2012. The answers to the questionnaire were analyzed descriptively. Since the data set collected in 2011 was structurally identical with the data set gained in 2007 both surveys were compared.

**Results: **Of the 24 companies represented on the vfa Clinical Research/Quality Assurance Subcommittee, 75% (18) took part in the survey. Survey evaluation was based on a total of 251 applications of these 18 companies submitted to 43 ECs. These account for about 21% of 1,214 applications for authorization of commercial and non-commercial phase I–IV clinical trials submitted to the regulatory authorities (BfArM and PEI) in 2011.

In comparison to 2007, a lower amount of applications (n=251 in 2011 vs. n=288 in 2007) was submitted to a slightly higher number of ECs (43 in 2011 vs. 40 in 2007). The amount of objections increased by 21% from 1,299 (2007) to 1,574 (2011) resulting in an average of 4.5 (2007) vs. 6.3 (2011) objections per application. Overall, the analysis of both formal and content related objections revealed almost the same pattern as in the previous survey. In total, the most frequent objections applied to the patient information and consent form followed in descending order by trial protocol content, miscellaneous, other application documents pursuant to § 7 (2) and (3) GCP-V, formal deficiencies pursuant to § 8 (1) GCP-V, and investigator and site qualifications. A trend towards a slightly increased rate of objections concerning patient information and consent form (+4%) and a minimal decrease in objections concerning investigator and site qualifications (–2%) was observed.

As in 2007, about 1 in 6 applications was still incomplete with formal objections. Whilst the proportion of study applications with objections related to the patient information and consent form (+7.2%), the trial protocol content (+11.6%), and documents according to § 7 (2) and (3) GCP-V (+11.8%) increased in 2011 compared to 2007, the amount of study applications with objections related to the investigator and site qualifications decreased by 6.3%.

**Conclusions:** The majority of findings with respect to quantity, quality and main focus of objections reported in the first survey in 2008 were also found in 2012, indicating a shared understanding of applicable measures and criteria by sponsors and ECs on how to ensure patient rights and well-being, data integrity, and high quality documentation in clinical trials.

## Zusammenfassung

**Ziel:** Das Ziel dieses Projektes war es, die Qualität und Quantität der Erstantragstellung auf zustimmende Bewertung einer klinischen Prüfung gemäß § 7 der deutschen GCP-Verordnung (GCP-V), die Qualität des Bewertungsverfahrens bei den Ethik-Kommissionen (EKs) in Deutschland sowie das Muster der Einwände seitens der EKs in ihren mit Gründen versehenen Bewertungen (Bescheide) zu evaluieren. Um etwaige Veränderungen über die Zeit zu identifizieren, wurden außerdem die Ergebnisse dieser Umfrage, mit den Ergebnissen einer in 2008 durchgeführten Umfrage verglichen.

**Methoden:** Auf der Grundlage von Bescheiden der für den Leiter der klinischen Prüfung jeweils zuständigen (federführenden) EK in 2011, wurde eine schriftliche Umfrage bei Mitgliedsunternehmen des Verbandes Forschender Arzneimittelhersteller e.V. (vfa) in 2012 durchgeführt. Die erhaltenen Antworten zu dem Fragebogen wurden deskriptiv ausgewertet. Da der in 2011 erhaltene Datensatz mit dem Datensatz in 2007 strukturell identisch war, wurden beide Umfragen miteinander verglichen.

**Ergebnisse: **Von den 24 Unternehmen, die im vfa-Unterausschuss Klinische Forschung/Qualitätssicherung vertreten sind, nahmen 75% (18) an der Umfrage teil. Der Auswertung dieser Umfrage liegen insgesamt 251 Antragsverfahren dieser 18 Unternehmen bei 43 EKs zugrunde. Diese stellen rund 21% der 1.214 bei den Bundesoberbehörden (BfArM und PEI) 2011 gestellten Anträge auf Genehmigung kommerzieller und nicht-kommerzieller klinischer Prüfungen der Phasen I–IV dar.

Im Vergleich zu 2007 wurden 2011 eine geringere Zahl von Anträgen (n=251 in 2011 vs. n=288 in 2007) bei einer geringfügig größeren Zahl an EKs (43 in 2011 vs. 40 in 2007) gestellt. Die Anzahl der Einwände stieg um 21% von 1.299 (2007) auf 1.574 (2011) und damit im Durchschnitt von 4,5 (2007) auf 6,3 (2011) Einwände pro Antrag. Insgesamt gesehen zeigt die Analyse der formalen und inhaltlichen Einwände ein Muster auf, das der vorangegangenen Umfrage sehr ähnlich ist. Die häufigsten Einwände betreffen die Patienteninformation und Einwilligungserklärung, in absteigender Reihenfolge gefolgt von Einwänden bezüglich Prüfplaninhalten, nicht-kategorisierende Einwände („Andere“), Einwände zu „Sonstigen Antragsunterlagen“ nach § 7 (2) und (3) GCP-V, Formmängel nach § 8 (1) GCP-V sowie Einwände, die sich auf Unterlagen zur Prüfer- und Prüfstellenqualifikation beziehen. Tendenziell zu beobachten war eine leicht erhöhte Rate von Einwänden bezüglich der Patienteninformation und Einwilligungserklärung (+4%) sowie eine geringfügige Abnahme von Einwänden, die sich auf Unterlagen zur Prüfer- und Prüfstellenqualifikation bezogen (–2%).

Wie in 2007 ist etwa jeder sechste Antrag unvollständig und weist formale Einwände zu den Antragsunterlagen auf. Während der Anteil der Studienanträge mit Einwänden bezüglich der Patienteninformation und Einwilligungserklärung (+7,2%), den Prüfplaninhalten (+11,6%) und „Sonstigen Antragsunterlagen“ nach § 7 (2) und (3) GCP-V (+11,8%) in 2011 im Vergleich zu 2007 zunahm, nahm der Anteil der Studienanträge mit Einwänden, die Unterlagen zur Prüfer- und Prüfstellenqualifikation betrafen, um 6,3% ab.

**Schlussfolgerungen: **Die Ergebnisse der ersten Umfrage in 2008 im Hinblick auf die Quantität, die Qualität und die Schwerpunkte der Einwände seitens der EKs, finden sich überwiegend auch in der Umfrage 2012. Dies zeigt ein übereinstimmendes Verständnis von Auftraggebern (Sponsoren) und EKs hinsichtlich der anzuwendenden Maßnahmen und Kriterien zur Sicherstellung der Rechte und des Wohlergehens der Patienten, der Datenintegrität und einer qualitativ hochwertigen Dokumentation in klinischen Prüfungen.

## Introduction

Clinical trials are important to improve and advance medical science and public health. A reliable partnership between patients, physicians, ECs, regulatory authorities, and sponsors is a prerequisite. For the conduct of clinical trials, the ECs have been proven to be an important element for assuring the rights, well-being, safety, and data protection of clinical trial subjects.

Clinical studies are very often conducted on a global scale. Taking competitive recruitment into account, high ethical standards combined with a timely review and approval process is a key element while setting up clinical studies in Germany. In order to support the tasks and responsibilities of ECs, seamless cooperation between ECs and sponsors is mandatory.

In 2008 a survey was performed by vfa to analyze the status of interactions between ECs and sponsors for the first time. Data showed that a major heterogeneity among ECs existed with respect to individual requirements and evaluations, in particular concerning patient information and consent documents as well as site and investigator qualifications. However, mutually agreed solutions were found in most cases of dissent between applicants and ECs by means of a constructive dialogue [1]. 

In order to follow-up on these findings and to verify the process of EC submission, evaluation, and EC-sponsor-interactions again, a second survey using the same questionnaire was performed 4 years later in 2012. Importantly, in these 4 years there were no legal changes in the clinical study regulatory environment. The legal framework is also described by Russ et al. in 2009 [1].

The conclusions drawn may provide evidence for both sponsors and ECs on how to further improve the quality of study applications and the harmonization of the EC review process, respectively.

## Methods

The present survey among all 44 member companies of the vfa was conducted from July to September 2012, based on reasoned opinions issued by the respective EC in charge of the coordinating principle investigator (coordinating EC) from January to December 2011.

**Figure 1:**
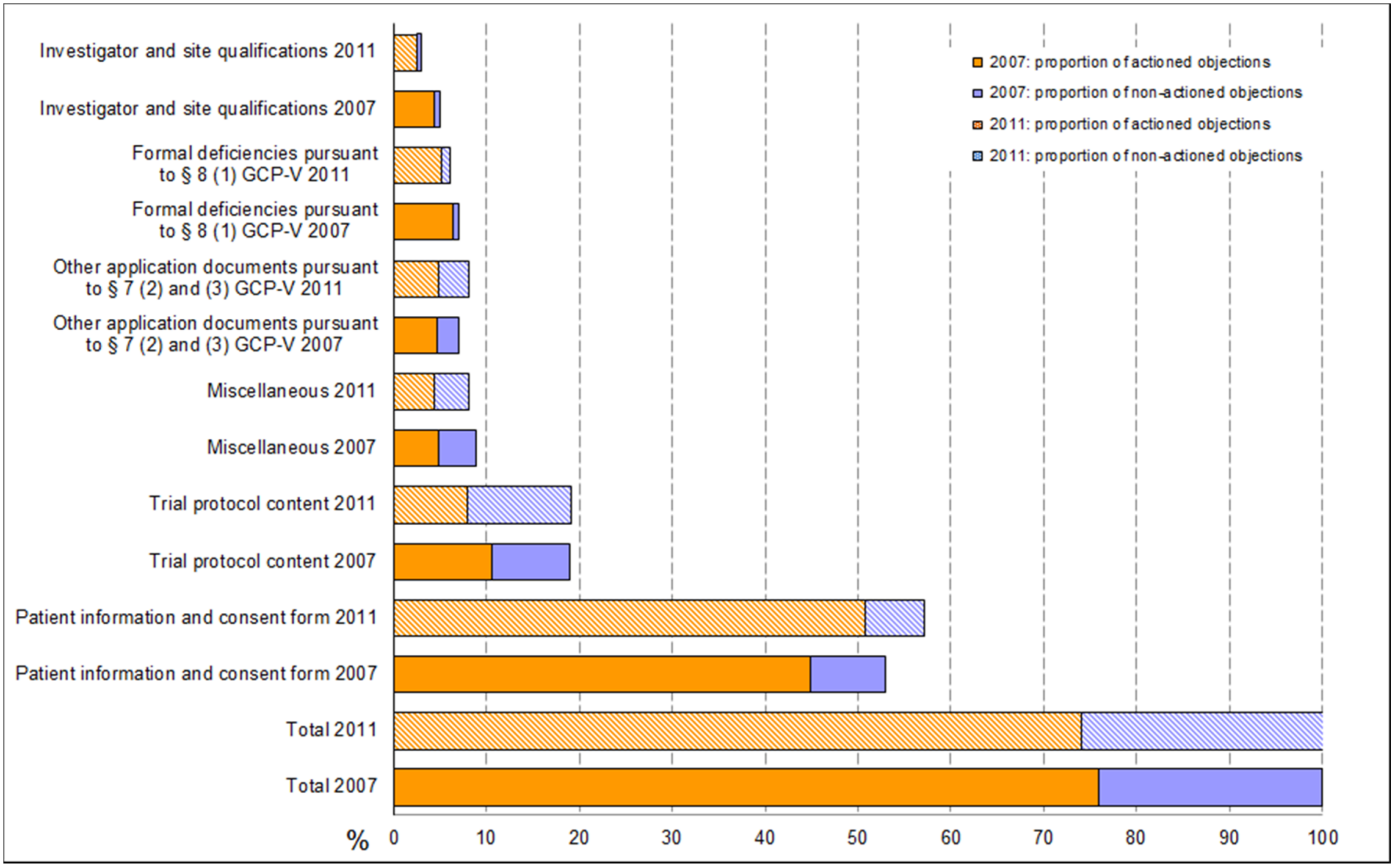
Breakdown of formal and content-related objections as a percentage of total objections (Phase I to IV) 2007 vs. 2011

The same questionnaire as for the first survey was used again (Attachment 1, Attachment 2). Among others, it captures the type and number of formal and content-related objections in the decisions of coordinating ECs after first application for a clinical trial pursuant to GCP-V § 7 (1–3) [2]. In detail, 10 questions asked for the number of initial applications, i.e. studies, the clinical trial phase, therapeutic indication, name of the respective coordinating EC, the number of formal and content-related objections per study application along with a brief description of the issue, the subjective evaluation and the applicant’s response to the objections, and whether or not the EC objection was challenged. The responding companies also classified the objections into 6 pre-specified evaluation categories. These were: Formal deficiencies pursuant to GCP-V § 8 (1) as well as 5 categories of content-related objections regarding patient information and consent document, trial protocol content, investigator and site qualifications, other application documents pursuant to GCP-V § 7 (2) and (3), and requests, remarks and recommendations not directly related to the application documents submitted pursuant to GCP-V § 7 (2) and (3), called “miscellaneous” here (see Figure 1 (Fig. 1)). As a slight variation to the first questionnaire, the second survey asked additionally, whether the sponsor company itself or a Clinical Research Organization (CRO) was acting as applicant.

Overall, the two surveys are based on similar data sets and were used for descriptive comparison. No formal statistics were pre-specified or applied.

In the following section, the data of the current investigation are presented in comparison to the first survey. The results of 2007 [1], are given in parentheses.

## Results

Of the 44 (44) companies organized in the vfa and included in the survey, 24 (22) companies were represented on the vfa Clinical Research/Quality Assurance Subcommittee. Eighteen (21) out of these 24 (22) companies took part in the survey. A total of 251 (288) applications for phase I–IV studies submitted by these 18 (21) companies to 43 (40) ECs in 2011 were evaluated. These account for about 21% of all 1,214 applications for authorization of commercial and non-commercial phase I–IV clinical trials submitted to the regulatory authorities (BfArM and PEI) in 2011 [3], [4].

A breakdown by clinical trial phase and therapeutic indication for the 251 evaluated applications is given in Table 1 (Tab. 1) and Table 2 (Tab. 2). No statistically significant difference (95% CI) was noted with respect to the category of the clinical trial phase (Table 1 (Tab. 1)) when comparing 2011 to 2007. A statistically significant increase in oncology trials from 17.3% (21.9%, 13.5%) in 2007 to 27.1% (32.6%, 22.1%) in 2011 was reported whereas the proportion of studies in all other therapeutic indications changed only slightly (Table 2 (Tab. 2)).

Out of the study applications analyzed, 88% were submitted by the sponsor or the legal representative of the sponsor in the EU. In 12% of cases CROs authorized by the sponsors made the application. From the 18 members of the vfa who participated in the survey 2012 (in 2008 the survey didn’t ask for the type of applicant), 9 companies performed the EC applications exclusively by themselves, 7 companies either performed the applications by themselves or delegated this task to CROs and only 2 companies had the EC submissions solely conducted by CROs.

### Frequency of formal and content-related objections in relation to total objections

Objections reported in reference to the patient information and consent document accounted for 57% (53%) of the total 1,574 (1,299) EC objections, followed by 19% (19%) objections related to the submitted trial protocol. Another 8% (7%) of total objections were due to formal deficiencies in other application documents according to GCP-V § 7 (2, 3) and 6% (7%) were due to formal deficiencies pursuant to GCP-V § 8 (1). Only 3% (5%) of total objections were in relation to specific documents on the site or investigators. Objections summarized under the categories of requests, remarks and recommendations not directly related to the application documents submitted pursuant to § 7 (2) and (3) (called “miscellaneous” here) contributed 8% (9%) (cf. Figure 1 (Fig. 1)).

Very similar to 2007, 26% (24%) of objections to the applications were reported by the vfa-members as having a lack of legal basis or it was claimed that the relevant information was already provided as part of the submission package, but had not been taken into consideration by the ECs. In such cases, the applicants made reference to the submitted documents or provided detailed explanations for not acting on the objections. The proportion of not mutually agreeable objections was highest in the categories of “Trial protocol content” 59% (44%), “Other documents pursuant to GCP-V § 7 (2) and (3)” 41% (33%), and “Miscellaneous” 46% (46%). Applicants acted on the majority of objections in the categories “investigator and site qualifications” and “patient information and consent document”, with response rates of 83% (87%) and 89% (85%), respectively.

### Frequency of formal and content-related objections in relation to study applications

**Figure 2:**
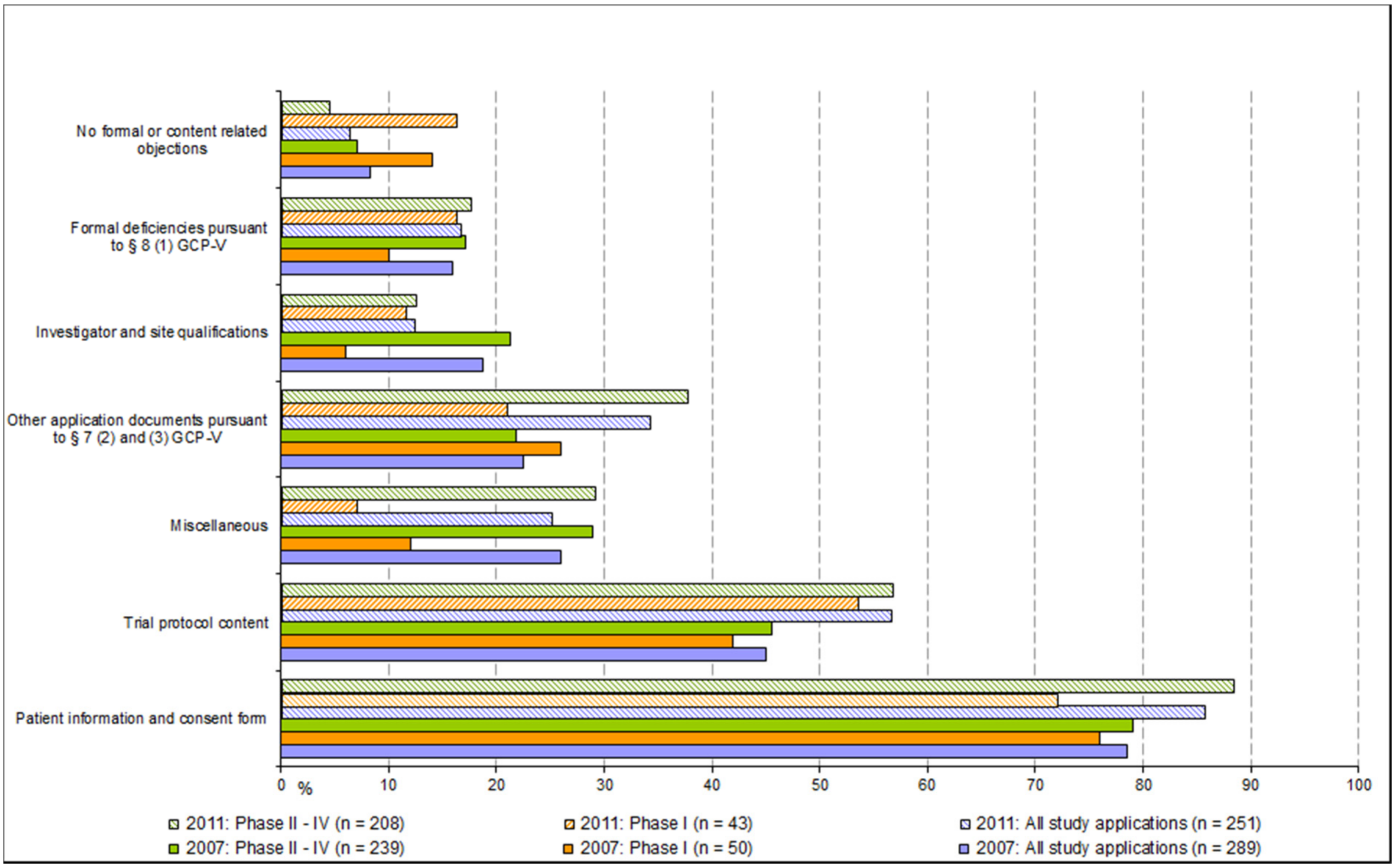
Study applications with formal and content-related objections in percent (Phase I to IV)

An analysis of study applications in terms of formal and content-related objections (cf. Figure 2 (Fig. 2)) shows that 85.7% (78.5%) of all study applications received at least one objection related to the submitted patient information and consent document. An amount of 56.6% (45.1%) of all study applications received at least one objection in terms of trial protocol content. The number of applications with objections to documents pursuant to § 7 (2) and (3) GCP-V increased from 22.5% in 2007 to 34.3% in 2011 whereas the number of applications with specific objections to investigator and sites qualifications decreased by 6.3% from 18.7% in 2007 to 12.4% in 2011. The proportion of incomplete applications with formal objections was 16.7% (16%) in this survey. Sixteen (24) out of the 251 (288) study applications (total 6.4% (8.3%) had neither formal nor content-related objections, while 4 (3) study applications were rejected on grounds of study design. The 10-day time limit for formal EC review was not missed (only 1 application in 2007). The ECs exceeded the 60-day processing time for content-based review for a total of 9 (8) applications (3.6% (2.8%)). No statistically significant difference (95% CI) in the frequency of objections in phase I study applications between 2007 and 2011 was observed. For phase II–IV study applications, only a statistically significant increase in objections concerning application documents pursuant to § 7 (2) and (3) GCP-V from 22% (95% CI: 27%, 17%) in 2007 to 37% in 2011 (43%, 31%) was detected. 

### Breakdown of study applications and breakdown of frequency of formal and content-related objections in relation to the ECs involved in the survey 

**Figure 3:**
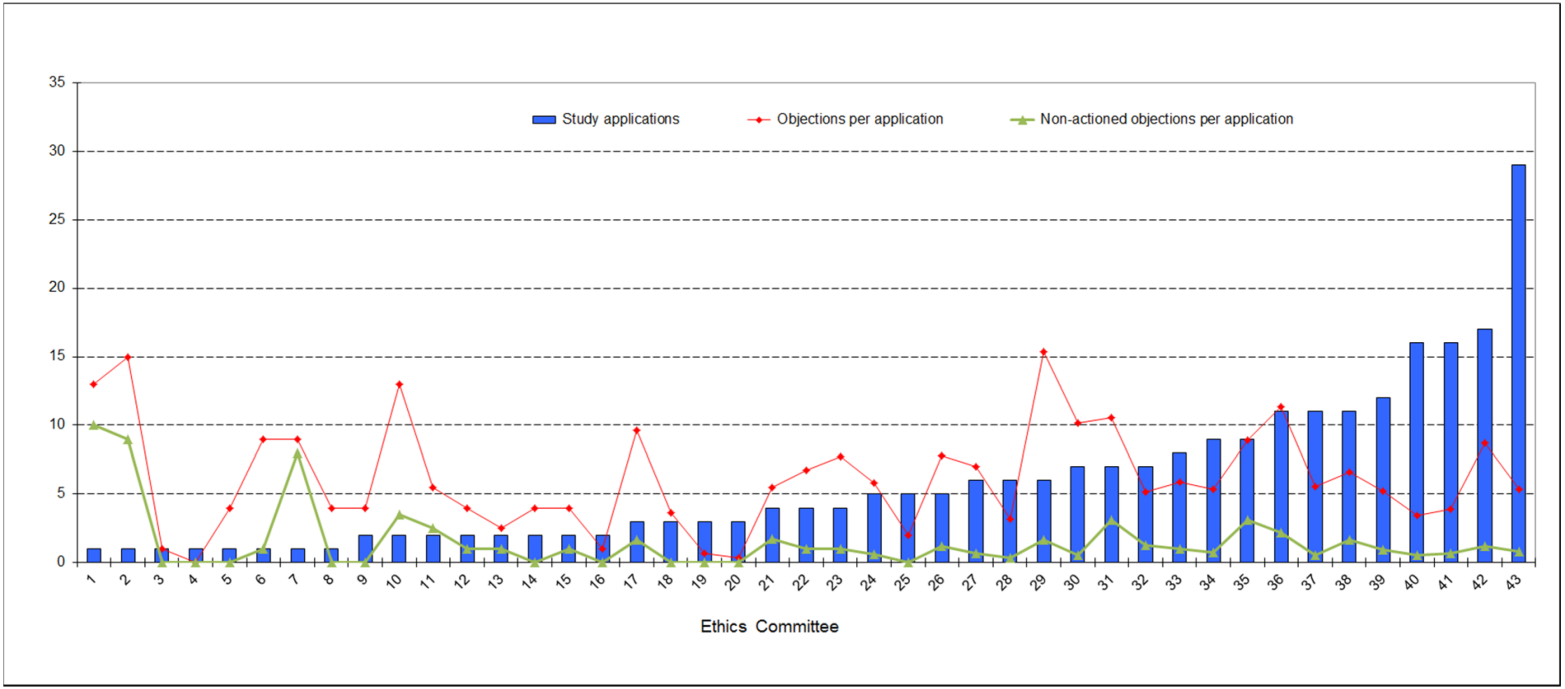
Number of study applications and number of objections per application; breakdown among the 43 ECs involved in the survey

A percentage of 48.2% (54.9%) of the 251 (288) study applications were submitted to 14 (14) ECs of state chambers of physicians and federal states. 51.8% (45.1%) were submitted to 29 (26) ECs of university hospitals. Only 8 (18.6%) of the total of 43 ECs involved in this survey each reviewed at least 10 applications, amounting to a total of 123 applications and hence accounting for almost half (49%) of all study applications. In 2007 10 (25%) of the total of 40 ECs were accounting for roughly half (53.8%) of all study applications. Figure 3 (Fig. 3) shows the number of study applications per EC.

Stratification of all ECs into two groups according to the number of applications reviewed by each (cf. Table 3 (Tab. 3)) shows that 60.5% (57.7%) of ECs reviewed only up to 5 study applications each, i.e. 25.1% (27.1%) of all study applications. These ECs on average raised less objections per application than the ECs in the group that each reviewed more than 5 applications. In 2007 the situation was contrary meaning all ECs especially those reviewing more than 5 applications were more homogeneous in their assessments of study applications. This EC group represents 39.5% (42.5%) of the 43 (40) ECs involved and reviewed 74.9% (72.9%) of the total 251 (288) study applications.

 The data also suggest a slightly different pattern of objections between ECs of state chambers of physicians and federal states on one hand and university hospitals on the other hand. About 60% of objections from ECs at university hospitals were in reference to the patient information and consent document compared to 53% from ECs of state chambers of physicians and federal states. Another 8% versus 4% of objections are related to formal deficiencies pursuant to GCP-V § 8 (1). Whereas 22% of objections from ECs at state chambers of physicians and federal states fell under the category trial protocol content opposed to 16% in that category from ECs at university hospitals.

**Figure 4:**
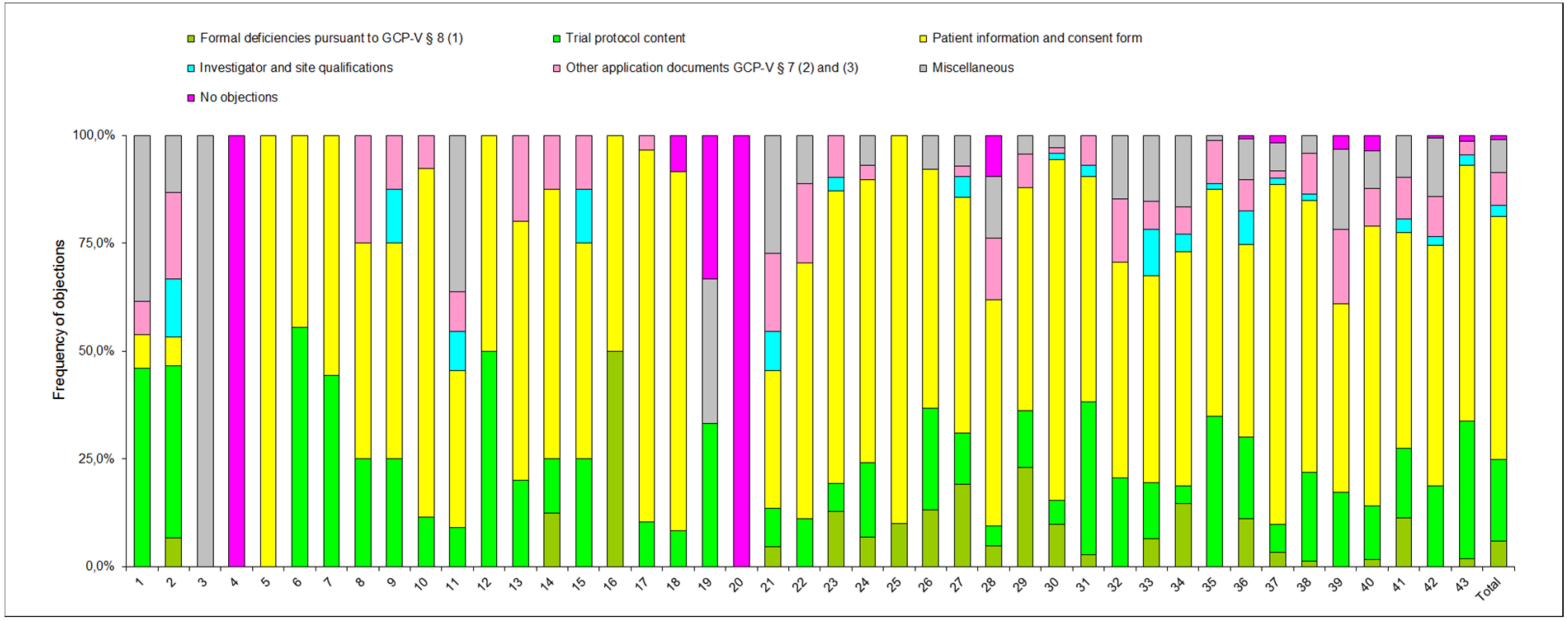
Frequency of objections in percent; breakdown among the 43 ECs involved in the survey

The pattern of objections by each individual EC is shown in Figure 4 (Fig. 4). The frequency distribution of the objections matched to the respective review categories (trial protocol content, investigator and site qualifications etc.) shows a very heterogeneous picture among the ECs. 

### Type of formal and content-related objections

Pursuant to GCP-V § 8, content-related review by an EC begins only after complete submission. If there are formal deficiencies, i.e. missing or incomplete documents pursuant to GCP-V § 7 (2) and (3), the applicant has 14 calendar days in which to address those deficiencies. The 30-day (for mono-center clinical trials) and 60-day time limit (for multi-center clinical trials) for content-related review of the study application begins after complete submission. Incomplete applications defer the review phase and prolong the overall procedure.

The formal and content-related objections are listed in descending order of frequency.

### Formal objections

These are the most common formal deficiencies pursuant to GCP-V § 8 (1):

- Missing or incomplete information on the qualifications of investigators/and or site suitability, e.g. missing proofs of qualifications (CVs, GCP certificate), inadequate description of patient recruitment process, and missing information about concurrent studies (GCP-V § 7 (3) 6. and 8.)
- Missing statement on information concerning possible financial and other interests of investigators in connection with the investigational medicinal products (GCP-V § 7 (3) 7.)
- Missing consent of hospital director or head of department to study conduct
- Missing CRF
- Missing sponsor confirmation that every investigator was informed of pharmacology/toxicology results by a scientist responsible for pharmacology/toxicology testing and the risks likely associated with the clinical trial (Drug Law § 40 (1) (7))

### Content-related objections

In keeping with the outcome of the survey, by far the most common content-related objections concern the submitted **patient information and consent documents**. Typical objections:

- Present patient information in language accessible to laypersons and explain jargon
- Present patient information with contact details and address of investigator/site, e.g. in the form of letterhead
- Present more clearly or add information on risks, adverse effects or side effects stating the percent frequency, based on the investigator brochure or package leaflet
- Provide more specific details about the pseudonymisation process in the data protection section
- Point out in the insurance cover section that the insurance terms are to be handed out to patients, and state clearly what the patient should do in the event of a possible injury/claim or emergency
- Clearly and precisely state the allowed birth control methods
- State that the primary care physician is to be informed of the patient’s study participation with the patient’s consent

Other content-related objections concerned the submitted **trial protocol**. The respondent companies cited the following items as being the most common requests in connection with trial protocol content:

- More precise description of existing inclusion/exclusion and/or additional inclusion/exclusion criteria
- Specific discontinuation criteria for the individual subject and for the entire clinical trial
- Justify the study design (placebo arm in particular), the scientific rationale, the sample size and/or the primary endpoint(s)

Objections relating to **investigator qualifications and site suitability** were reported as follows:

- Investigator qualifications:
  - Incomplete CVs (e.g. missing information about number of studies previously conducted)
  - Inadequate proof of existing GCP knowledge; mention of the need for a Drug Law/GCP-V course
  - Information missing on other medical staff involved in the study.

- Site suitability:
  - Requests for hospital director or head of department’s consent to study conduct.
  - Missing information on concurrent studies and any measures to ensure recruitment.

Typical objections concerning **application documents pursuant to GCP-V § 7 (2) and (3***)* were:

- Requests to modify terms and conditions of the patient insurance or to provide study-specific insurance certificate despite the submission of appropriate patient insurance certificate pursuant to the Drug Law § 40 (§ 7 (3) 13.))
- Provide clarification regarding remuneration and publication in the sponsor-site agreement
- Adjust wording of advertisement for patient recruitment
- Adjust wording of patient emergency card

A large number of requests, remarks and recommendations not directly related to the application documents to be submitted pursuant to § 7 (2) and (3) GCP-V were summarized in the **Other objections** category. The most common were:

- Recommendation (or demand, in some cases) to take out accident en-route insurance
- Demand to ensure patient data protection (i.e. pseudonymization and anonymization, respectively) when collecting personal related data (e.g. no full date of birth or double coding for genetic testing)
- Request for clarifications and recommendations related to application documents pursuant to GCP-V § 7 (2) and (3)

## Discussion

The obtained results from 2 comparable surveys within a time period of 4 years are based on 21% of all applications for authorization of commercial and non-commercial phase I–IV clinical trials submitted to the regulatory authorities (BfArM and PEI) in 2007 [4], [5] and 2011 [3], [4], respectively and therefore might be deemed to be representative. It cannot be ruled out that the small differences observed may be partly due to structural differences between the time points. A full statistical analysis could not be applied to all parameters due to confidentiality of some data. The results may be viewed from different perspectives, i.e. what do the data reveal for the process of performing clinical studies in Germany, what is the consequence and learning for the sponsors and ECs for clinical trials conducted in Germany and last but not least what is the impact on the rights and well-being of patients in clinical trials.

### Patients in clinical research – what has changed?

For patients it’s important to understand the research question and the benefits and risks of their study participation. This includes understanding the patient information, the content of the informed consent, and data protection aspects. With respect to the documents most relevant for patients, ECs are focusing on patient information and consent form. More than 50% of all objections, slightly increased from 53% (2007) to 57% (2011), are related to this topic. Also, objections related to data protection seem to play an increasingly important role. By critically reviewing study applications with respect to protecting patients, ECs are ensuring that patient rights, well-being and data privacy are addressed appropriately. From the surveys it seems that a trustful cooperation on a professional level between sponsors and ECs has developed over the years to safeguard patients and to allow for innovative research in unmet medical needs.

### The clinical research environment

The more reliable and standardized the EC procedures the better Germany’s position as an attractive research location. Thus, the vfa is interested in insights how the EC process is perceived by companies and interested in how to learn and improve.

The amount of studies as well as the focus of indications has changed within 4 years. In comparison to 2007, a lower amount of applications (n=251 in 2011 vs. n=288 in 2007) was submitted to ECs. This is in line with data from both federal regulatory authorities in Germany BfArM and PEI [4], [5]. The amount of objections increased from 1,299 (2007) to 1,574 (2011), resulting in an average of 4.5 (2007) vs. 6.3 (2011) objections per application.

One reason for the increase in objections could be due to the fact that more complex studies, such as in i.e. oncology may require more intense review and a trend to more scientific input was observed in addition. Related to the trial protocol content, the focus of objections moved from more formal requests (e.g. to explain the method of contraception, to add the publication policy, SAE report address and monitoring details) to enquiries challenging the scientific rationale, the sample size and/or the primary endpoint. The increase in objections may also be due to intensified evaluation of patient related documents like patient information and consent documents as well as advertisement for patient recruitment.

### Performance of ECs

In 2007 about 25% of 40 ECs reviewed 54% of applications while in the recent survey 18.6% of 43 ECs reviewed 49% of applications. Together with the observation that 40% of the 43 ECs in this survey assess roughly 75% of all applications, this could be seen as a trend towards concentration to specific ECs. This may also reflect a shift to more complex studies and certain indications. More scientifically challenging studies with innovative drugs in unmet medical needs may have to be conducted in specialized clinics and university hospitals. Thus, specific ECs allocated to these centers may get more applications consequently. This is also shown in an increase of applications submitted to ECs at university hospitals by almost 7% in 2011.

Similar to the previous survey the timelines for review (formal & content) were almost always kept, indicating reliability of EC processes and adherence to stipulated timeframes.

### Sponsor – EC Interaction 

From the surveys, it seems that a shared understanding between sponsors and ECs has developed over the years. With respect to the frequency of formal and content related objections almost the same pattern was found in 2007 and 2011. However, the frequency of objections related to patient information and consent form, trial protocol, and § 7 increased considerably while objections to site and investigator quality dropped. The later observation could be attributed to some lessons learnt by sponsors and investigators while ECs harmonized their requirements on documents to support the qualification of site and study personnel.

### Areas for improvement

As observed in 2007, substantial heterogeneity in terms of requirements and evaluations, in particular with respect to patient information and consent document as well as application documents pursuant to GCP-V § 7 (2) and (3) still exists in 2011.

From the applicants’ point of view, these requirements seem to be partly subjective in many cases rather than based on definite legal specifications. Also similar to 2007 about one quarter of objections lack legal basis or the requested data were already included in the submitted document package. However, constructive solutions were found in most cases of dissent between applicant and EC which enable both the implementation and justified non-implementation of actions on the objections in question.

## Conclusions

The present survey on clinical trial review procedures by ECs based on the experiences of the pharmaceutical companies represented in the vfa confirms both the distribution pattern and the type of formal and content related objections from the first survey. In line with current trends in the industry, more complex studies for unmet medical needs are conducted. 

The majority of findings with respect to quantity, quality and main focus of objections reported in the first survey in 2008 were also found in 2012, indicating a shared understanding of applicable measures and criteria by sponsors and ECs on how to ensure patient rights and well-being, data integrity, and high quality documentation in clinical trials.

Although high level of expertise is seen in objections, high variance among ECs is notable. While patient information and consent form related objections remain number one priority, a wide variety of objections is noticed, depending on the individual EC. To further improve the dialogue between sponsors and ECs an increased alignment among ECs would be beneficial.

## Notes

### Data

The underlying data are available upon request from the first author.

### Conflict of interest

The authors are employees of vfa member companies.

### Acknowledgements

The authors wish to thank the vfa member companies that participated in the survey, and Dr. F. Hundt and Dr. T. Ruppert for critical review of the manuscript.

## Supplementary Material

Questionnaire page 1 – Explanations and instructions

Questionnaire page 2 – Data collection sheet

## Figures and Tables

**Table 1:**
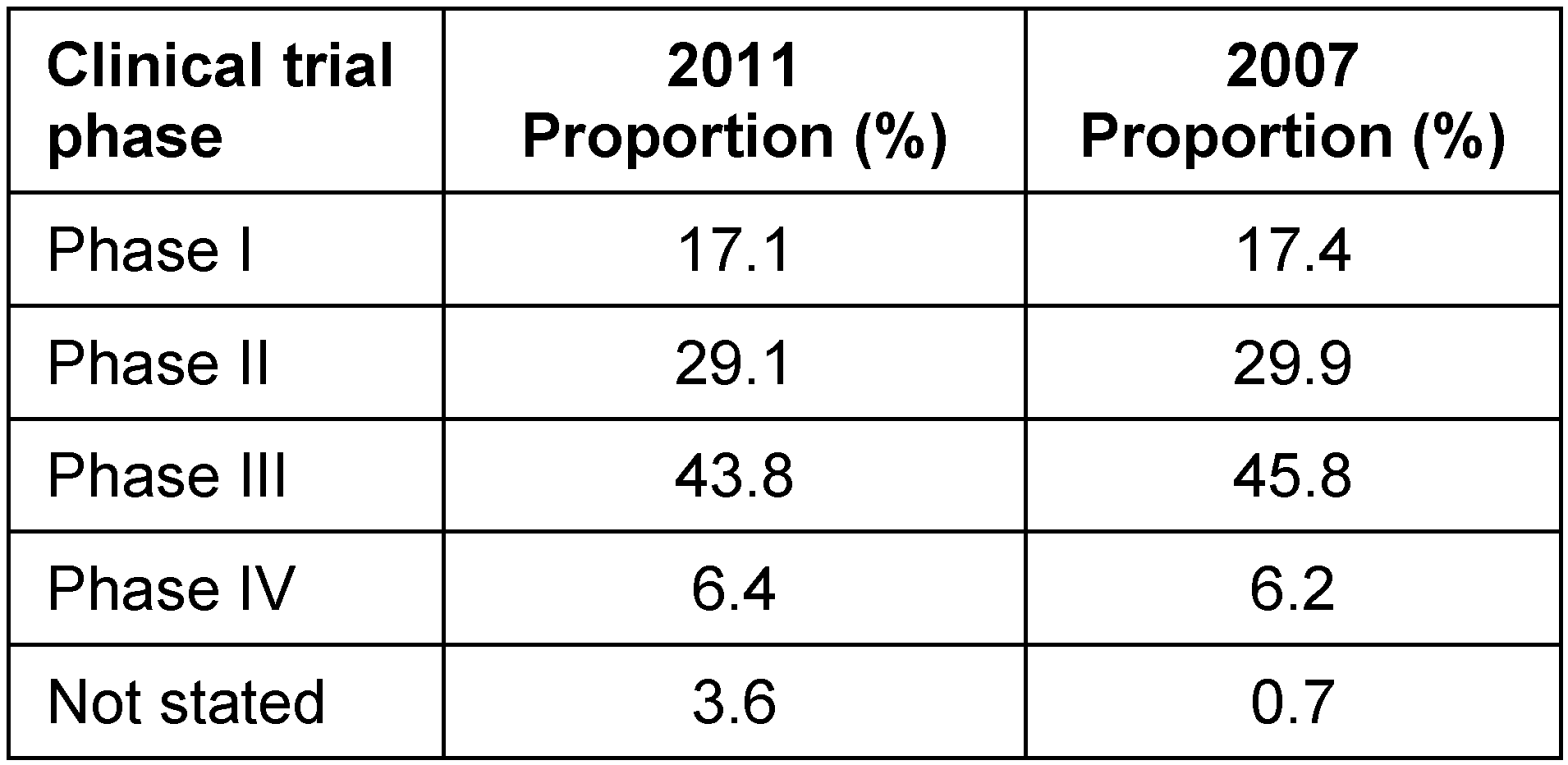
Breakdown of evaluated applications by clinical phase I–IV in 2011 and 2007

**Table 2:**
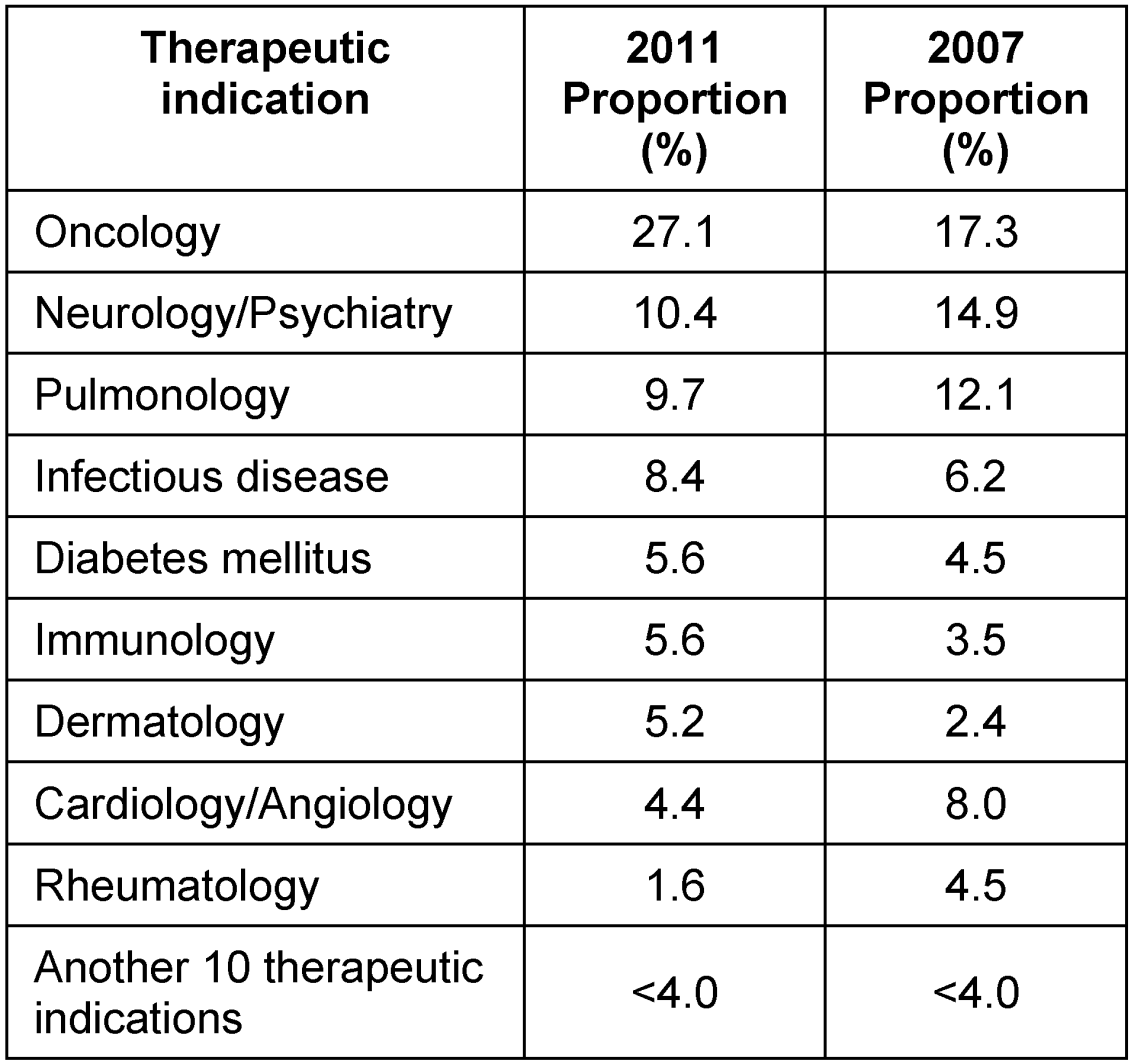
Breakdown of evaluated applications by therapeutic indication in 2011 and 2007

**Table 3:**
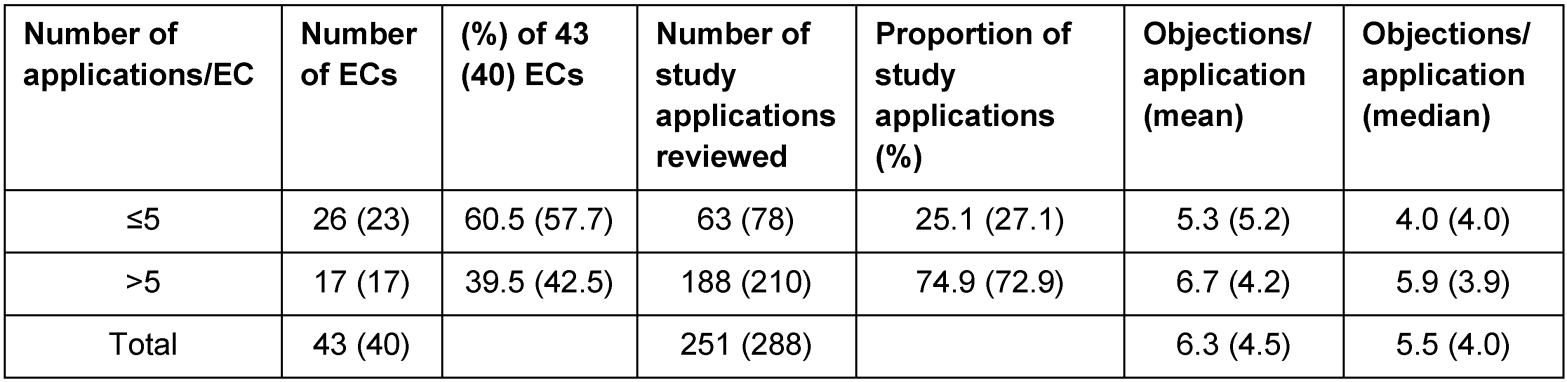
Number of study applications and number of objections per application; breakdown into 2 groups of ECs involved in the survey in 2011; figures from the survey in 2007 are in parenthesis
