# Supplementary material for: Evaluation of clinical trials by Ethics Committees in Germany – results and a comparison of two surveys performed among members of the German Association of Research-Based Pharmaceutical Companies (vfa)
Source: Ger Med Sci. 2015 Jan 27;13:Doc02. doi: 10.3205/000206 (PMC4311050; doi:10.3205/000206)
Supplement: Questionnaire page 1 – Explanations and instructions [file GMS-13-02-s-001.pdf]

Questionnaire Page 1 - Explanations and instructions

What information will be collected

The type and frequency of formal and content-related objections to first applications according to § 7 of the German Good Clinical Practice Regulation (GCP) ordinance (German: GCP-Verordnung, GCP-V) issued by the respective coordinating ethics committee will be documented. Formal and content-related objections to subsequent changes of a clinical trial according to § 10 GCP-V issued by the respective coordinating ethics committee will not be documented. Recommendations and advices will be documented only if they cannot be ignored and a statement by the sponsor is required.

Period of data collection

First applications dating from January to December 2011

Legend

Serial number of the study

The serial number defines the study in the data collection

Ethics Committee

The following classification as prefix to the name of the ethics committee (EC) has to be used
LA = EC of federal state (Ethik-Kommission des Landes)
LÄK = EC of state chamber of physicians (Ethik-Kommission der Landesärztekammer)
UK = EC at university hospital (Ethik-Kommission der Medizinischen Fakultät einer Universität (Universitäts-Klinikum))
TU = EC at technical university (Ethik-Kommission der Medizinischen Fakultät einer Technische Universität)

Serial number of the objections per study

The serial number defines the number of objections per study

Evaluation category

Please classify the objection into 1 of the following categories

1. Formal deficiencies pursuant to GCP-V § 8 (1) (e.g. CV and other investigator qualification document not complete)
2. Trial protocol content (e.g. design, rationale, statistics)
3. Patient information and consent document / form
4. Investigator and site qualifications
5. Other documents pursuant to GCP-V § 7 (2) and (3)
6. Non-adherence of the ethics committee to the timeline for the formal review of the application
7. Non-adherence of the ethics committee to the timeline for the content-related review of the application
8. Miscellaneous
9. No objections

Study phase

Please select the respective study phase number I - IV (1 - 4) from the drop down list

Indication

Please select the respective indication from the drop down list

1. Endocrinology
2. Psychiatry and Neurology
3. Oncology
4. Urology and Nephrology
5. Infectious disease
6. Cardiology and angiology
7. Immunology
8. Dermatology
9. Metabolic disorder
10. Gastroenterology
11. Hematology
12. Pneumology
13. Gynecology and andrology
14. Rheumatology
15. Pain
16. Orthopedics
17. Otolaryngology
18. Intensive care
19. Diabetology
20. Others

Comments

Please describe your response to the objection of the ethics committee, especially the reason in case of not acting on the objection as well as the ethics committee's decision in each case (e.g. ethics committee accepted or didn't accept sponsor's not acting on the objection). Sponsor's subjective evaluation / comments on the objection or further information are important to better understand the issue

Attachment 1 to: Russ H, Busta S, Jost B, Bethke TD. Evaluation of clinical trials by Ethics Committees in Germany – results and a comparison of two surveys performed among members of the German Association of Research-Based Pharmaceutical Companies (vfa). GMS Ger Med Sci. 2015;13:Doc02.
DOI: 10.3205/000206, URN: urn:nbn:de:0183-0002064
