# Supplementary material for: Evaluation of clinical trials by Ethics Committees in Germany – results and a comparison of two surveys performed among members of the German Association of Research-Based Pharmaceutical Companies (vfa)
Source: Ger Med Sci. 2015 Jan 27;13:Doc02. doi: 10.3205/000206 (PMC4311050; doi:10.3205/000206)
Supplement: Questionnaire page 2 – Data collection sheet [file GMS-13-02-s-002.pdf]

Questionnaire Page 2 - data collection sheet

Formal and content-related objections of coordinating ethics committees to first applications for clinical trials pursuant to § 7 (1 – 3) of the German Good Clinical Practice Regulation

Company no. (serial)	Study no. (serial)	Ethics Committee	Objections per study (serial number)	Objection / Request (brief description of the issue, na = not applicable if no objection)	Evaluation Category	Study phase	Indication	Application submitted by sponsor/legal representative or Clinical Research Organization	Acted on objection (yes, no)	Comments

Attachment 2 to: Russ H, Busta S, Jost B, Bethke TD. Evaluation of clinical trials by Ethics Committees in Germany – results and a comparison of two surveys performed among members of the German Association of Research-Based Pharmaceutical Companies (vfa). GMS Ger Med Sci. 2015;13:Doc02.
DOI: 10.3205/000206, URN: urn:nbn:de:0183-0002064
